# Hypothermic Storage of 3D Cultured Multipotent Mesenchymal Stromal Cells for Regenerative Medicine Applications

**DOI:** 10.3390/polym14132553

**Published:** 2022-06-23

**Authors:** Irena Vackova, Eliska Vavrinova, Jana Musilkova, Vojtech Havlas, Yuriy Petrenko

**Affiliations:** 1 Department of Biomaterials and Tissue Engineering, Institute of Physiology of Czech Academy of Science, 14220 Prague, Czech Republic; irena.vackova@fgu.cas.cz (I.V.); jana.musilkova@fgu.cas.cz (J.M.); 2 Department of Neuroregeneration, Institute of Experimental Medicine of the Czech Academy of Sciences, 14220 Prague, Czech Republic; eliska.vavrinova@iem.cas.cz; 3 Department of Orthopaedics and Traumatology, Second Faculty of Medicine, Charles University, 15006 Prague, Czech Republic; vojtech.havlas@lfmotol.cuni.cz

**Keywords:** multipotent mesenchymal stromal cells, Wharton’s jelly, Hyalofast^®^, hypothermic storage, paracrine activity

## Abstract

The regulatory requirements in cell processing, in the choice of a biomaterial scaffold and in quality control analysis, have to be followed in the clinical application of tissue-engineered grafts. Confirmation of sterility during quality control studies requires prolonged storage of the cell-based construct. After storage, preservation of the functional properties of the cells is an important prerequisite if the cells are to be used for cell-based tissue therapies. The study presented here shows the generation of 3D constructs based on Wharton’s jelly multipotent mesenchymal stromal cells (WJ-MSCs) and the clinically-acceptable HyaloFast^®^ scaffold, and the effect of two- and six-day hypothermic storage of 3D cell-based constructs on the functional properties of populated cells. To study the viability, growth, gene expression, and paracrine secretion of WJ-MSCs within the scaffolds before and after storage, xeno-free culture conditions, metabolic, qPCR, and multiplex assays were applied. The WJ-MSCs adhered and proliferated within the 3D HyaloFast^®^. Our results show different viability of the cells after the 3D constructs have been stored under mild (25 °C) or strong (4 °C) hypothermia. At 4 °C, the significant decrease of metabolic activity of WJ-MSCs was detected after 2 days of storage, with almost complete cell loss after 6 days. In mild hypothermia (25 °C) the decrease in metabolic activity was less remarkable, confirming the suitability of these conditions for cell preservation in 3D environment. The significant changes were detected in gene expression and in the paracrine secretion profile after 2 and 6 days of storage at 25 °C. The results presented in this study are important for the rapid transfer of tissue engineering approaches into clinical applications.

## 1. Introduction

Multipotent mesenchymal stromal cells (MSCs) are currently the focus of many studies seeking novel approaches for the treatment of various diseases. It has already been confirmed that the main mechanism of the therapeutic action of MSCs is based on their paracrine activity. MSCs secrete a cocktail of growth factors, cytokines, extracellular vesicles, or mitochondria, which target the host cells and improve their functional activity [1,2,3]. Being isolated from various human tissues, umbilical cord Wharton’s jelly (WJ) is a unique source of MSCs with high proliferation potential and paracrine activity [4,5]. We recently compared the growth kinetics, the immune properties, and the paracrine activity of MSCs derived from human adult tissues (bone marrow, BM, and adipose tissue, AT) and WJ (fetal adnexa tissue), and we demonstrated a significantly higher expansion rate of WJ-MSCs compared to BM- and AT-MSCs, while maintaining the neuroprotective and immunomodulatory activity of these cells in vitro [4]. Moreover, given the absence of ethical issues related to source tissue collection, the use of WJ-MSCs is considered promising for allogeneic transplantation, with approximately 60 clinical trials being registered at clinicaltrials.gov accessed on 25 May 2022.

The secretome profile of MSCs is highly dependent on the microenvironment. Currently, many attempts are being made, searching for clinically-acceptable pre-treatment strategies for enhancing the paracrine activity of cells [6,7,8,9]. Switching the cell culture conditions from a classical 2D environment to a 3D environment has been shown to improve the key therapeutic properties of MSCs [6,8]. The self-organization of MSCs into spheroidal aggregates has been shown to improve the immunomodulatory and anti-inflammatory properties, via activation of anti-inflammatory TNFα-stimulated gene/protein 6 (TSG-6) and prostaglandin E2 (PGE-2) secretion [7,8]. The expression of angiogenic factors (Vascular Endothelial Growth Factor A, VEGF-A, basic Fibroblast Growth Factor, FGF-2 or Hepatocyte Growth Factor, HGF) is also enhanced in spheroid cultures, along with anti-apoptotic factor B-cell lymphoma 2 (BCL-2) [10,11]. The embedding of MSCs into biomaterial hydrogels and scaffolds has been shown to provide similar cell activation [12,13,14]. The encapsulation of MSCs into collagen l-hyaluronic acid hydrogels resulted in a significant increase in the secretion of proangiogenic, neuroprotective, and immunomodulatory paracrine factors [12]. The 3D culture of MSCs in microporous hydrogels enhanced the paracrine factor secretion activity [13]. 3D cultures of MSCs can therefore be considered promising for the development of therapeutic grafts with enhanced paracrine secretion activity, able to induce and support the regenerative processes of the target tissues.

The choice of the carrier may play a crucial role not only in the success of MSC activation but also in the rate of transfer into clinical practice. Among biomaterials, hyaluronic acid (HA) holds a strong position, being naturally involved in many biological processes such as cell differentiation and tissue repair [15,16]. Hyaluronan polymer-based scaffolds are at the forefront of clinical tissue engineering, and many HA-based products are currently available for clinical applications. In our study, we applied commercially available HyaloFast^®^ (Anika Therapeutics, Inc. Padova, Italy), a non-woven biodegradable scaffold composed of HYAFF-11^®^, a semisynthetic insoluble polymer obtained by the esterification of all carboxyl groups of HA with benzyl alcohol, certified as a class III medical device (Directive 93/42/EEC). As a cell-free medical device, HyaloFast^®^ is intended for the repair of chondral or osteochondral lesions; however, several reports have been published on the use of HyaloFast^®^ in combination with MSCs in animal [17] and human studies [18]. We suggest that the use of clinically-approved products may significantly accelerate the clinical application of cell-seeded 3D grafts.

The highly regulated manufacturing process of 3D tissue-engineered grafts for further implantation should not only ensure high therapeutic activity of the product but should also meet safety requirements. The generated products must be thoroughly evaluated for the absence of any viral and bacterial contamination. The final sterility tests should therefore be prepared prior to packaging of the final product, and there should be no subsequent handling; otherwise, sterility may be compromised, and the tests would need to be repeated [19,20]. According to European Pharmacopoeia, the microbiological control of cellular products requires 7–14 days of incubation using aerobic and anaerobic growth media and two incubation temperatures [21]. However, several assays have already been approved by the U.S. Food and Drug Administration (FDA) that allow the time of the analysis to be reduced to 3–5 days (BacT/ALERT^®^ (bioMérieux, Nürtingen, Germany), BACTEC™ (Becton Dickinson, New Jersey, USA), and Rapid Milliflex^®^ (Merck Millipore, Darmstadt, Germany). It has recently been shown that the BacT/ALERT^®^ 3DTM Dual T system (bioMérieux) allows sufficient and trustworthy results to be generated during 5 days of incubation [21]. However, even in the case of rapid sterility evaluation, prolonged storage of the finally packed preparation represents an additional challenge. Current cryopreservation strategies allow successful preservation of cell suspensions, but in the case of 3D scaffold-based systems the typical cell viability post-thaw comprises around 50 % [22,23,24,25]. Such viability rates are insufficient for achieving the optimal therapeutic outcomes. The storage of 3D MSC-based grafts under hypothermic conditions is an alternative strategy for short-term graft preservation. It is crucial to choose an optimal storage temperature that is able to preserve the function of the cells at maximum levels.

The aim of our study was to generate 3D allogeneic grafts based on WJ-MSCs and HyaloFast^®^ non-woven scaffolds under xeno-free conditions, and to study the effect of short-term storage under mild hypothermia (25 °C) and under strong hypothermia (4 °C) on the viability, the paracrine secretion profile, and the gene expression of 3D cultured cells.

## 2. Materials and Methods

### 2.1. Tissue Collection and Source of Chemicals

Discarded human umbilical cords (UC) were obtained from healthy neonates after spontaneous delivery at the University Hospital in Pilsen (Czech Republic). All donors provided written informed consent before UC collection. The human UCs were donated anonymously. All studies involving human tissues or cells were approved by the Ethical Committee of the Institute of Experimental Medicine Academy of Sciences Czech Republic, Prague. All methods were performed in accordance with the relevant guidelines and regulations. Unless otherwise indicated, chemicals were purchased from Sigma-Aldrich (St Louis, MO, USA).

### 2.2. Cell Culture

Umbilical cord collection, WJ-MSC isolation and characterization have already been described in our previous publications [4,26]. The cells were cultured in a xeno-free complete culture medium (CCM) consisting of α-minimal essential medium (αMEM; LONZA, Basel, Switzerland), 5% pooled human platelet lysate (PL; Bioinova, Ltd., Prague, Czech Republic), and 10 μg/mL gentamicin (Sandoz, Holzkirchen, Germany). The cells were cultured at 37 °C in a humidified atmosphere containing 5% CO_2_ with regular media changes twice a week. After reaching 90% of confluence, the cells were trypsinized using 0.05% Trypsin/EDTA (Life Technologies, Carlsbad, CA, USA), were counted, and were used for seeding the scaffolds.

### 2.3. Scaffolds, Cell Seeding, and Culture

The commercially-available ready-to-use 3D product HyaloFast^®^ (Anika Therapeutics inc.) was used as a scaffold for this study. The scaffold was sterilely cut into 0.5 × 0.5 cm squares in a laminar box, placed in bacterial dishes (P-Lab, Prague, Czech Republic) with a non-adherent surface (five squares in each), and immersed in CCM for 20–30 min to hydrate the scaffold. Cell seeding was performed by immobilizing the cells with a blood plasma-based hydrogel. Briefly, 300,000 cells were resuspended in 29.7 μL human blood plasma (Thomayer University Hospital, Prague, Czech Republic), 0.8 μL 10% CaCl_2_ (HBM Pharma, Martin, Slovak Republic), and 2.5 μL human serum (Biowest, Nuaillé, France), and were pipetted onto one moistened scaffold. The 3D constructs were left to polymerize for 5–10 min and were then cultured in 10 mL CCM in a CO_2_ incubator at 5% CO_2_ and 37 °C for 8 days. The culture medium was changed twice a week.

### 2.4. Hypothermic Storage of 3D Constructs

After 8 days of culture, the 3D constructs were transferred to sterile screw-cap cryotubes (Nunc, Roskilde, Denmark) with 1 mL fresh CCM, one construct per tube, and the cell-seeded constructs were incubated at 37 °C and 5% CO_2_ with the lid remaining ajar to allow air exchange and saturation of the medium with CO_2_. After 1 h of incubation, the cryovials were tightly sealed and were stored at either 4 °C or 25 °C for 2 or 6 days.

### 2.5. Resazurin Assay

Cell metabolic activity, proliferation, and recovery of WJ-MSCs after storage in 3D constructs were assessed using the resazurin assay [27]. To evaluate the cell growth within the scaffold, the metabolic activity was measured repeatedly on days 1, 4, and 7 in three identical randomly selected samples and was measured on day 8 completely in all samples. The post-storage recovery was measured after 2 or 6 days of hypothermic storage at 4 °C and 25 °C. Each sample was labelled to allow pairing of pre- and post-storage measurements. Briefly, the samples were transferred to a new 24-well plate containing 0.5 mL of fresh CCM with 40 µM resazurin in each well. The samples were incubated for 2 h at 37 °C in a humidified atmosphere with 5% CO_2_. Then, 150 µL of the solution was transferred to a 96-well plate, and the fluorescence (Ex/Em = 530/590) was measured on a Synergy^TM^ HT Multi-Mode Microplate reader (BioTek, Santa Clara, CA, USA). The measured values were corrected for the background control (CCM with resazurin). Each experiment was performed in triplicate.

### 2.6. Immunofluorescent Staining

To visualize the cells in the scaffold, the cell-seeded constructs were stained with an antibody against cytoskeletal protein vimentin on days 1 and 7 of culture. Monoclonal mouse antibody VI-10 specific to vimentin (11-460-C100, dilution 1:200 in PBS; Exbio Prague, CR) and goat anti-rabbit IgG (H+L) antibody Alexa FluorTM Plus 488 (A32723, dilution 1:400; Thermo Fisher Scientific Inc., Waltham, MA, USA) were used as the primary and secondary antibody, respectively. The cell nuclei were counterstained with bisBenzimide H33258 (B1155, 10 μg/mL). The cells were evaluated under a confocal microscope (Stellaris 8, Leica Microsystems, Wetzlar, Germany).

### 2.7. Total RNA Isolation

Total mRNA was isolated from the 3D constructs using the Animal Tissue RNA Purification Kit (Norgen Biotek, Ontario, Canada). Due to the small amount of mRNA in one 3D construct, three HyaloFast^®^-cell constructs were pooled into a single experimental sample. Samples were solubilized twice (repeatedly) in 200 µL of RL buffer. Isolation was then carried out according to the manufacturer’s protocol. The amounts of RNA were quantified using a NanoDrop™ OneC Spectrophotometer (Thermo Fisher Scientific). Reverse transcription was performed using the Omniscript Reverse Transcription Kit (205113; Qiagen, Hilden, Germany) using random hexamers (New England Biolabs, Inc, Ipswich, MA, USA).

### 2.8. Real-Time PCR

Real-time PCR was performed to investigate the changes in the relative mRNA expression of 15 genes selected to perform the multi-sided screening of different cell functions (Table 1) in samples isolated before and after 2 and 6 days of storage.

The mRNA level was quantified using 5x HOT FIREPol^®^ Probe qPCR Mix Plus (ROX) (08-36-00001; Solis BioDyne, Tartu, Estonia) and by TaqMan Gene Expression Assays (4331182; Thermo Fisher Scientific), labelled with the FAM reporter dye specific to the following human genes: *CXCL12* (Hs00171022_m1), *CCL2* (Hs002341410_m1), *ICAM* (Hs00164932_m1), *VCAM* (Hs01003372_m1), *TWIST* (Hs01675818_s1), *PDL1* (Hs00204257_m1), *COX2* (Hs00153133_m1), *TGFB1* (Hs00998133_m1), *IL6* (Hs00174131_m1), *VEGFA* (Hs00900055_m1), *HGF* (Hs00300159_m1), *FGF2* (Hs00266645_m1), *P21* (Hs01040810_m1), *HIF1A* (Hs00153153_m1), and *BAX* (Hs00180269_m1), with *GAPDH* (Hs00187842_m1) as the reference gene. The experiments were performed using LightCycler^®^ 480 System Technology (Roche, Basel, Switzerland) in a 96-well optical reaction plate. The qPCR was carried out in a final volume of 20 µL. All amplifications were run under the same cycling conditions: 12 min at 95 °C, followed by 42 cycles of 15 s at 95 °C and 1 min at 60 °C. The data for each experimental point are the mean of 4 measurements of 2 independent samples. The relative gene expression was calculated as 2^−ΔΔCt^, and the results are presented as the log2-fold change. The results are related to the reference gene and to the non-stored cell samples (d 0) as a control. The results are expressed as Mean ± SEM.

### 2.9. Conditioned Medium (CM) Production and Proteomic Analysis

After 2 and 6 days of storage at 25 °C, immediately after the resazurin assay, the cell-seeded constructs were transferred to 1 mL of fresh CCM and were incubated at 37 °C/5% CO_2_. After 24 h, the conditioned medium was collected and was stored at −80 °C until use. The concentrations of the growth factors and cytokines (SDF-1α, VEGF-A, VCAM-1, FGF-2, HGF, MCP-1, IL-6, and bNGF) were determined using the Luminex^®^-based multiplex ProcartaPlex^®^ Immunoassay (Thermo Fisher Scientific). Nine samples were measured for each time interval. CMs collected on day 8 from the same samples that were later measured after 2 and 6 days of storage at 25 °C served as controls. The measurement was performed on a Bio-Plex 200 Instrument (Bio-Rad, Prague, Czech Republic) according to the manufacturer’s instructions. All samples were analyzed in duplicate. The cytokine and growth factor concentrations (pg/mL or ng/mL) in the samples were derived from measured MFIs using fitted standard curves.

### 2.10. Statistical Analysis

Statistical analyses were performed using SigmaStat (Systat Software, San Jose, CA, USA). Multiple-comparison procedures were conducted by analysis of variance, using the Student-Newman-Keuls method. *p* ≤ 0.05 was considered significant.

## 3. Results

### 3.1. Growth and Paracrine Activity of WJ-MSCs within 3D HyaloFast^®^

**Figure 1:**
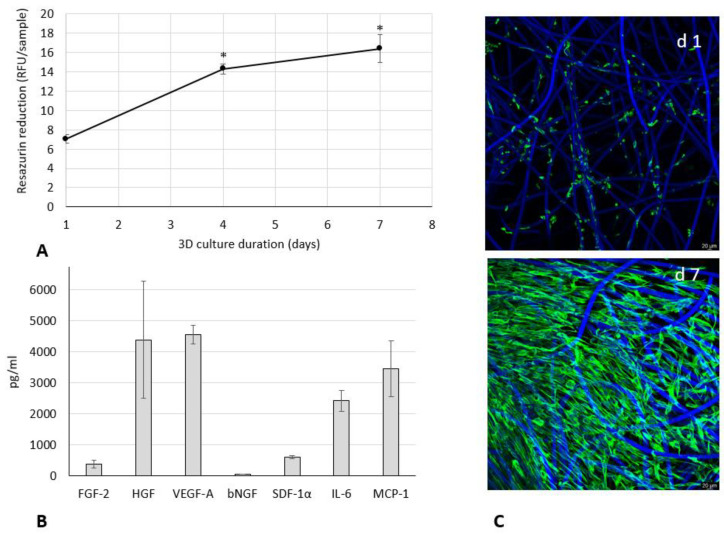
Growth (**A**), paracrine secretion profile (**B**), and morphology (**C**) of WJ-MSCs during culture in 3D HyaloFast^®^ scaffolds (*n* = 8). (**A**)—resazurin assay; (**B**)—multiplex ProcartaPlex^®^ Immunoassay of conditioned medium samples (Mean ± SD). (**C**)—Vimentin (green)/bisBenzimide (blue) staining of cells at d 1 (upper) and d 7 (lower) of culture in standard conditions; *—the data are significantly (*p* < 0.05) different compared to d 1 of culture.

The proliferation of WJ-MSCs within HyaloFast^®^ was assessed by resazurin assay (Figure 1A).

After 4 days of 3D culture, a remarkable two-fold increase in resazurin activity was observed, confirming the growth of cells within the scaffold. During the subsequent culture, the rate of cell proliferation slowed down, likely due to density-dependent growth inhibition. The complete population of the scaffold by WJ-MSCs was confirmed by a microscopic evaluation (Figure 1C). To confirm the paracrine secretion activity of the WJ-MSCs within 3D HyaloFast^®^, the content of growth factors and cytokines was assessed in a conditioned medium collected after 24 h of culture (Figure 1B). We detected a high content of HGF, VEGF-A, and MCP-1 (in the range of 3.5–4.6 ng/mL), a lower amount of IL-6 (2.4 ng/mL), and a low content of FGF-2, bNGF, and SDF-1α (less than 1 ng/mL). Thus, 7 days of culture of WJ-MSCs in HyaloFast^®^ allowed the generation of a viable 3D graft with remarkable paracrine activity.

### 3.2. The Effect of Hypothermic Storage on the Recovery of WJ-MSCs in 3D Conditions

**Figure 2:**
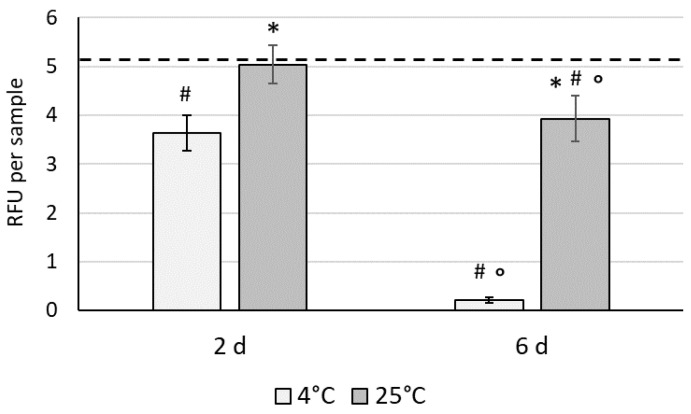
The effect of hypothermic storage on the metabolic activity of WJ-MSCs within 3D HyaloFast^®^ scaffolds (*n* = 5 in triplicate); resazurin assay, the solid line represents the non-stored control; Data are presented as Mean ± SD; #—data are significantly (*p* < 0.05) different compared to the control (before storage) group; *—data are significantly (*p* < 0.05) different compared to the 4 °C group; °—data are significantly (*p* < 0.05) different compared to same group on day 2.

To select the optimal temperature for hypothermic storage of 3D constructs based on WJ-MSCs within HyaloFast^®^, the samples were placed in sealed vials and were stored at 4 °C and 25 °C for 2 and 6 days. The metabolic activity was determined for the constructs by resazurin assay, and the data were presented as mean relative fluorescence units (RFU) per each sample (Figure 2).

After 2 days of storage, a significant decrease in metabolic activity of the WJ-MSCs was detected in the 4 °C group (3.63 ± 0.36 RFU) compared to the 25 °C group (5.03 ± 0.39 RFU). After 6 days of storage, a dramatic drop in cell viability was detected in the 4 °C group (0.2 ± 0.05 RFU), whereas a less remarkable decrease was observed in the samples stored at ambient temperature (25 °C, 3.93 ± 0.47 RFU). The storage of cells under mild hypothermia (at 25 °C) is therefore superior to strong hypothermic storage, and further studies were focused on the 25 °C group.

### 3.3. The Effect of Mild Hypothermic Storage under Ambient (25 °C) Conditions on the Gene Expression and Paracrine Secretion Profile of WJ-MSCs within 3D HyaloFast^®^

**Figure 3:**
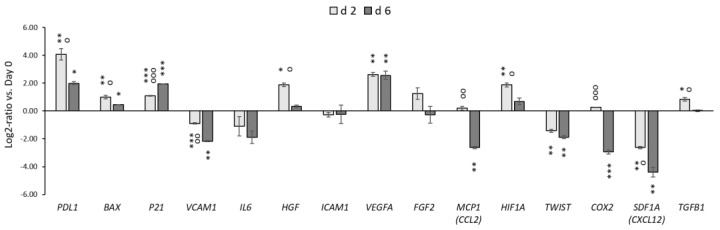
The effect of hypothermic storage on the relative gene expression of selected genes in WJ-MSCs cultured within 3D HyaloFast^®^, assessed by qPCR (*n* = 4). The graph shows the log2-fold changes of the ΔΔCt values of the indicated genes in comparison to day 0. The data are presented as Mean ± SEM. *, **, ***—the data are significantly different compared to non-stored cells (***—*p* < 0.001; **—*p* < 0.01; *—*p* < 0.05); °, °°, °°°—the data are significantly different between the d 2 group and the d 6 group (°°°—*p* < 0.001; °°—*p* < 0.01; °—*p* < 0.05).

Before and after storage, we analyzed the expression of 15 genes related to the function: chemokines (*SDF1A*/*CXCL12*, *MCP1*/*CCL2*), adhesion molecules (*ICAM*, *VCAM*), transcription factors (*TWIST*), immunoregulatory/inflammatory/anti-inflammatory (*PDL1*, *COX2*, *TGFB1*, *IL6*), angiogenic/neurotrophic factors (*VEGFA*, *HGF*, *FGF2*), the genes associated with cell cycle, metabolism, and apoptotic processes (*P21*, *HIF1A*, and *BAX*) (Figure 3).

After 2 days of hypothermic storage, we detected significant upregulation of the *PDL1*, *BAX*, *P21*, *HGF*, *VEGFA*, *TGFB1*, and *HIF1A* genes. Simultaneously, the expression of *COX2*, *SDF1A* (*CXCL12*), *MCP1* (*CCL2*), *TWIST*, *VCAM*, *ICAM*, and *IL6* genes was downregulated or unchanged. The duration of storage also affected the expression of separate genes. Here, the expression of *PDL1*, *HGF*, *MCP1* (*CCL2*), *HIF1A*, *VCAM1*, *COX2*, *SDF1A*, and *TGFB1* was significantly reduced after 6 days compared to 2 days of storage. Conversely, the expression of *P21* was increased after prolonged storage.

**Figure 4:**
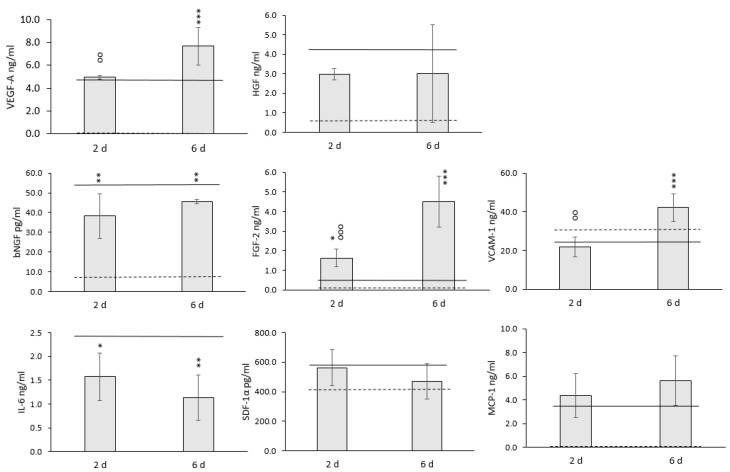
The secretion profile of 3D cultured WJ-MSCs after hypothermic storage (*n* = 6). The data are presented as Mean ± SD. Solid line—non-stored cells, dashed line—background control (the medium without cells). *, **, ***—the data are significantly different compared to the non-stored cells (***—*p* < 0.001; **—*p* < 0.01; *—*p* < 0.05); °°, °°°—the data are significantly different between the 2d group and the 6d group (°°°—*p* < 0.001; °°—*p* < 0.01).

The effect of hypothermic storage on the paracrine activity of WJ-MSCs within HyaloFast^®^ is shown in Figure 4.

No significant changes in the levels of MCP-1, HGF, and SDF-1α were detected in the conditioned medium of 3D cultured WJ-MSCs after 2 and 6 days of storage. A significant decrease was indicated for bNGF content. However, even in the control group, the values did not exceed 100 pg/mL. A significant decrease was detected for IL-6 for both storage periods (Figure 4). However, a significant increase in the content of VEGF-A, FGF-2, and VCAM-1 was indicated after hypothermic storage. The levels of these growth factors became even higher after 6 d of storage. Based on the obtained data, we may conclude that short-term storage of 3D WJ-MSC grafts did not have any detrimental effects on the paracrine secretion capacity of the cells.

## 4. Discussion

In this study we have shown the generation of a 3D paracrine construct based on WJ-MSCs and HyaloFast^®^ using xeno-free clinically-acceptable conditions, and we propose a way to preserve the viability and the paracrine properties of the cells for a period of 6 days, as required to complete sterility testing. We chose HyaloFast^®^ as a 3D scaffold due to its high applicability in clinical applications, and also its confirmed biocompatibility and biodegradation [17,18,28,29]. HyaloFast^®^ is based on a semisynthetic insoluble polymer obtained by the esterification of all carboxyl groups of HA with benzyl alcohol (HYAFF-11^®^). It was previously shown that HYAFF-11^®^ provides an appropriate environment, supporting growth, migration, proliferation, and differentiation of MSCs into chondrogenic lineage [30,31,32]. Successful fabrication of tissue-engineered osteochondral grafts has been shown for MSCs loaded into a HYAFF-11^®^ sponge [33]. The bone marrow concentrate grown within the HYAFF11^®^ scaffolds was able to differentiate into chondrogenic [34] and osteogenic [35] lineages.

As a cellular component, WJ-MSCs were considered optimal for fast generation of the 3D graft due to the high proliferation rate and the strong paracrine secretion capacity, as shown in our previous study [4]. In the present study, we confirmed the proliferation of WJ-MSCs in a 3D environment and complete population of HyaloFast^®^ during 7–8 days of culture. The overall process of cell seeding, complete population of the scaffold, and subsequent storage for quality control analysis took a total of 14 days. We detected high levels of angiogenic factors HGF, VEGF-A, as well as MCP-1 and IL-6 responsible for the immunomodulatory activity of MSCs in the conditioned medium obtained from the 3D cultures [36].

In this study, we used blood plasma-based hydrogel to improve the seeding efficiency and immobilize the WJ-MSCs within the HyaloFast^®^. It is widely accepted that the 3D culture of MSCs in fibrin-based hydrogels supports the proliferation, differentiation, and paracrine activity of cells (reviewed in [37,38]). In our previous study, we found remarkable increase of FGF-2 and VEGF secretion by adipose tissue MSCs, cultured in human blood plasma-based hydrogels [39]. Therefore, we believe that the high paracrine secretion of WJ-MSCs within the HyaloFast^®^ occurs via the combinatorial action of 3D environment, composed of HyaloFast^®^ and human plasma-based hydrogels. The fast manufacturing times and the high paracrine activity would be promising for wide application of the proposed 3D WJ-MSC/HyaloFast^®^ grafts in allogeneic applications.

As mentioned before, the clinical application of cell-based grafts requires the completion of sterility testing of the final packed product, and the storage conditions may significantly affect the therapeutic properties of the graft. It is known that a decrease in temperature slows down the metabolic processes in cells, and the hypothermic storage approach may allow the state of the cells in the pre-storage stage to be preserved. Therefore, in the second part of our study we compared two temperature regimes (4 °C and 25 °C) for the 2 and 6 days of hypothermic storage of the generated 3D constructs. We found that storage at ambient, mild hypothermic conditions (25 °C) was superior to storage at 4 °C. The metabolic activity of cells stored under strong hypothermic conditions for a period of 48 h decreased significantly, whereas at the ambient temperature (25 °C), the metabolic activity remained almost unchanged. Subsequent storage of cells at 4 °C until day 6 resulted in the complete loss of cell viability within the scaffolds, confirming that 25 °C is the temperature of choice during hypothermic storage of 3D grafts.

It is known that irreversible changes in cellular structures may occur at 2–8 °C and may lead to cell apoptosis. In cell suspensions, profound hypothermia causes perturbation of ion balances, leading to osmotic cell swelling, oxidative stress, and an extracellular and intracellular pH switch [40,41]. We have previously shown that the storage of bone marrow MSC suspensions in a conventional saline medium led to a dramatic drop in cell viability after 48 h at 4 °C. In this case, only the use of specially-designed hypothermic solutions could improve the cell viability [42]. However, little is known about the effects of hypothermic preservation on cells populated within 3D scaffolds. Although we have shown that alginate encapsulation improves the viability of MSCs after short-term storage [43], the cells were not attached and were distributed in alginate beads as an entrapped suspension. Tam et al. showed high viability of induced pluripotent stem cell-derived MSCs within a tissue-engineered bone graft [44]. Another report showed poor osteogenic capacity of tissue-engineered bone preserved in an osteogenic medium at 4 °C [45]. Lower survival of MSCs at 4 °C compared to 25 °C has been shown for encapsulated cell aggregates [46]. We showed around 90% recovery of WJ-MSCs after 6 d storage at 25 °C, which we believe to be clinically-acceptable values. Further study was directed towards a deeper evaluation of the effect of mild hypothermic storage on cell characteristics - gene expression and the paracrine secretion profile.

We have not observed a remarkable decrease in the content of most of the growth factors and cytokines analyzed in our study. Only significantly lower secretion of bNGF and IL-6 was detected after both storage periods. Interestingly, however, the secretion of FGF-2 and VEGF-A remarkably increased after 6 d of storage at 25 °C. The secretion of high FGF-2 amounts is not a typical feature of MSCs, and before the storage, the FGF-2 content in the conditioned medium comprised only around 250 pg/mL. Therefore, the remarkable increase in FGF-2 secretion was probably connected with processes that occurred during storage. It is known that the prolonged storage of cells and tissues below physiological temperatures results in the moderation of metabolic activity and cell cycle deceleration or the initiation of apoptotic processes [47]. In this study, the significant upregulation of *P21* was detected after 2 and 6 days of storage, indicating the possible temperature-induced cell cycle arrest. Vice versa, it was shown previously that FGF-2 release might lead to the upregulation of *P21* [48]. The upregulation of *P21* expression may also correspond to the initiation of apoptotic processes in cells within the 3D construct [49]. It was recently shown that an increase in FGF-2 release could be associated with the initiation of apoptotic processes within the cells [50,51]. In our study, we have indicated the upregulation of pro-apoptotic *BAX*, which aligns with these data and can affect FGF-2 release. The apoptotic nature of the enhanced paracrine activity of MSCs has been recently proposed in several studies [52,53]. In parallel, the *VEGF* signaling is closely connected with hypoxia and the expression of *HIF1A*, and the significant upregulation of the *HIF1A* gene during storage may explain the increase in VEGF-A secretion [54]. Interestingly, the expression of *PDL1* involved in the immunomodulatory activity of MSCs was significantly upregulated after storage, and the *PDL1* expression can also be associated with the increased expression of *HIF1A* [55]. Conversely, the level of IL-6 in the conditioned medium decreased significantly after storage, which was accompanied by downregulation of the *IL6* gene (Figure 3 and Figure 4). Although many reports have been focused on evaluating the effect of profound hypothermia (at 4 °C) on different types of cells and tissues, little is currently known about the exact mechanisms of cell responses, which occur in MSCs during the storage at moderate hypothermia.

The data therefore suggests that hypothermic storage may affect the gene expression and the paracrine secretion, however more studies are needed to reveal the exact mechanisms of these changes.

## 5. Conclusions

We have shown a clinically-acceptable procedure for the fast generation of a paracrine 3D graft based on WJ-MSCs and HyaloFast^®^ for applications in regenerative medicine. We have further evaluated the effect of hypothermic storage of 3D cell-based constructs on the functional properties of populated cells, and we have shown the different viability of cells after storage under mild hypothermia (25 °C) and under strong hypothermia (4 °C). At 25 °C, the viability of the WJ-MSCs was significantly higher than at 4 °C. We then indicated the changes of gene expression of WJ-MSCs after storage, and we observed the changes in the paracrine profile of the cells. The results presented in this study are important for accelerating the transfer of tissue engineering approaches towards clinical applications.

## Figures and Tables

**Table 1:** Evaluated genes and abbreviations.

Gene Symbol	Full Gene Name
***Chemokines:***	
*CXCL12/SDF1a*	C-X-C Motif Chemokine Ligand 12/SDF1AStromal Cell-Derived Factor 1
*CCL2/MCP1*	C-C Motif Chemokine Ligand 2/MCP1Monocyte Chemoattractant Protein 1
***Adhesion molecules:***	
*ICAM1*	Intercellular Adhesion Molecule 1
*VCAM1*	Vascular cell adhesion protein
***Transcription factors***:	
*TWIST*	Twist Family BHLH Transcription Factor
*IL6*	Interleukin 6
***Immunoregulatory*/*inflammatory*/*anti-inflammatory genes:***	
*PDL1*	Programmed death-ligand 1
*COX2*	Cyclooxygenase 2
*TGFB1*	Transforming Growth Factor Beta 1
*IL6*	Interleukin 6
***Angiogenic/neurotrophic factors:***	
*VEGFA*	Vascular Endothelial Growth Factor A
*HGF*	Hepatocyte Growth Factor
*FGF2*	Fibroblast Growth Factor 2
***Genes associated with cell cycle, metabolism, and apoptotic processes:***	
*P21*	Cyclin-Dependent Kinase Inhibitor 1
*HIF1A*	Hypoxia Inducible Factor 1 Subunit Alpha
*BAX*	BCL2 Associated X Protein

## Data Availability

The data is available from the corresponding author on request.
